# Feasibility and acceptability of a joint employee-manager workplace intervention to support mental health and job retention (MENTOR 2.0)

**DOI:** 10.1186/s12889-025-24457-7

**Published:** 2026-07-18

**Authors:** Arianna Prudenzi, Olivia Hastings, Libby Grey, Naomi Johnston, Jonathan Stuart, Steven Marwaha

**Affiliations:** 1 Institute for Mental Health, School of Psychology, University of Birmingham, Birmingham, B15 2TT UK; 2 School of Psychology, The University of Sheffield, Sheffield, S1 4DP UK; 3 Mind Charity, 2 Redman Place E20 1JQ, London, E20 1JQ UK; 4 Birmingham and Solihull Mental Health Foundation Trust, Birmingham, B13 8QY UK

**Keywords:** Workplace intervention, Wellbeing, Mental health, Burnout, Productivity, Employees, Managers

## Abstract

Employees experiencing mental health difficulties frequently encounter barriers to both securing and sustaining employment. Despite increased recognition of mental health in the workplace, most interventions continue to target employees in isolation, with limited attention to the relational and organisational contexts in which mental health issues arise. To address this gap, we developed MENTOR 2.0 - the first joint employee–manager workplace intervention designed to enhance employee wellbeing and job retention through targeted improvements in work engagement, interpersonal relationships at work, and psychological flexibility. Building on our initial feasibility study (MENTOR 1.0) (Prudenzi et al., 2024, https://doi.org/10.1016/j.jcbs.2023.100720), MENTOR 2.0 involved four individual and one joint session with employees and their line managers, facilitated by a trained Mental Health Employment Liaison Worker (MHELW) - a novel role designed to facilitate conversations between employees and line managers. Twenty-three employees and nineteen managers participated in the study. All completed baseline, mid-intervention, and post-intervention surveys, along with 18 brief daily diaries per participant. Feasibility outcomes were positive: 94% of planned sessions were delivered as scheduled, the average programme duration was 65 days, and post-intervention retention was 87%. Qualitative feedback indicated high acceptability, with participants valuing the opportunity for open and confidential dialogue with managers, as well as the supportive role of the MHELW. Preliminary quantitative findings suggested reductions in psychological distress and improvements in productivity, psychological flexibility, work engagement, and workplace interpersonal functioning. While the single-arm design and limited sample size constrain generalisability, the study followed rigorous feasibility trial methods and demonstrates a novel, context-sensitive intervention model with clear translational potential. MENTOR 2.0 advances current practice by embedding mental health promotion within the social dynamics of the workplace, shifting away from individualised treatment models toward a more relational and systemic approach.

Feasibility trial registration MENTOR: ISRCTN79256498 (20/09/2021).

**Supplementary Information:**

The online version contains supplementary material available at https://doi.org/10.1186/s12889-025-24457-7.

## Introduction

Poor mental health in the workplace is a major public health concern in the UK. The prevalence of mental health difficulties among the workforce is alarming [1, 2], with mental ill-health accounting for an estimated 28% of the total burden of disease in the UK - compared to 16% each for cancer and heart disease [3]. The economic impact is substantial, with mental health problems estimated to cost UK employers between £33 billion and £42 billion annually [4, 5], primarily through increased absence, staff turnover, burnout, and reduced productivity [4]. In addition, there is increasing concern about the wider implications for organisations, workplace health systems, compensation authorities, and social welfare infrastructure [6].

Workplace factors can contribute to a range of mental health issues, including stress, depression, anxiety, and burnout [7, 8]. These challenges are often exacerbated by long working hours, limited social support, and the stigma associated with disclosing mental health problems [9, 10]. Disclosure is frequently perceived as risky, and employees may avoid seeking support due to fears of stigma or discrimination. The role of line managers is critical in addressing and promoting mental health, as they are central to creating psychologically safe and inclusive workplace cultures [5, 6]. Managers who are trained to recognise mental health issues and respond supportively can positively influence team wellbeing [11, 12]. Open communication, proactive support, and the implementation of mental health-promoting policies all contribute to healthier work environments and improved retention.

Despite increased awareness of these challenges, there remains a persistent knowledge gap regarding how to effectively implement workplace mental health interventions that engage both employees and their managers. In response, we developed MENTOR, a multifaceted intervention combining individual and joint sessions aimed at educating both parties, building a supportive workplace culture, providing coping strategies, facilitating access to further support, and enhancing psychological resilience. Grounded in the principles of Contextual Behavioural Science (CBS) [13], MENTOR represents an innovative alternative to established vocational models such as Individual Placement Support (IPS).

While IPS is a well-established, evidence-based model focused on helping individuals - often with severe mental illness - enter employment through rapid job search, CV support, and employer engagement, MENTOR differs in several important ways. It is designed for individuals already in employment and seeks to support them in sustaining their roles and improving their wellbeing. Rather than emphasising placement, MENTOR applies principles of CBS to enhance psychological flexibility, values-based action, and interpersonal functioning within the existing workplace setting.

Two key innovations of the MENTOR model are:

- Its delivery by a Mental Health Employment Liaison Worker (MHELW), and,.
- The inclusion of joint employee–manager sessions to strengthen interpersonal relationships and communication at work.

In this feasibility study, we evaluated a refined version of the intervention (MENTOR 2.0), building on the findings from our initial feasibility trial [1, 13]. The study was preregistered on AsPredicted (#134200) on 14/12/2023. Consistent with a feasibility trial design, the intervention was offered to employees regardless of formal mental health diagnosis. The primary aims were to assess the feasibility and acceptability of the updated MENTOR 2.0 model and to conduct preliminary evaluations of its potential impact on employee mental health and work-related outcomes.

The preregistered aims of MENTOR 2.0 were as follows:

Aim 1: To assess the acceptability and feasibility of delivering the MENTOR 2.0 intervention in a real-world workplace context.

Aim 2: To explore whether MENTOR 2.0 was associated with improvements in state psychological health outcomes (perceived distress, mood, job satisfaction, wellbeing, work engagement, and productivity), and state psychological processes (psychological flexibility, mental health awareness, interpersonal relationships at work, work engagement).

Aim 3: To examine whether these relationships were moderated by initial levels of psychological distress and/or the presence of a self-reported mental health condition at baseline.

 Aim 4: To determine whether the quality of the employee-manager relationship improved from baseline to post-intervention.

These aims were preregistered on AsPredicted (Registration ID: #134200; 14/12/2023), and no changes were made following preregistration. The primary objective of this study was to assess feasibility and acceptability, with secondary analyses exploring potential signals of impact. As such, findings related to effectiveness should be interpreted cautiously and are intended to inform the design of a future, adequately powered trial.

## Method

### Design

This feasibility study employed an interval-contingent repeated-measures design. Participants were asked to complete a series of self-report surveys at multiple time points: baseline, mid-intervention, and post-intervention survey. In addition to these time-point surveys, participants completed a total of 18 evening daily surveys over the course of the intervention period. These included three consecutive baseline daily surveys administered prior to the start of the intervention, followed by 15 daily surveys distributed after each of the five intervention sessions (three per session). This design enabled the monitoring of short-term, session-linked changes in both psychological outcomes and hypothesised psychological processes over time, providing granular data on participant experience and response trajectories. The primary purpose of this design was to assess the feasibility and acceptability of the intervention delivery and evaluation procedures, rather than to determine its definitive effectiveness.

The intervention consisted of five sessions, delivered by a trained Mental Health Employment Liaison Worker (MHELW) affiliated with Burton and District Mind, a local mental health charity. For a description of the role of the MHELW. For further details on the MHELW role and intervention components, please refer to the study protocol [13].

Ethical approval was granted by the Science, Technology, Engineering and Mathematics Ethical Review Committee at the University of Birmingham (Approval Date: 17/02/2023, Reference: ERN_22-1335).

### Recruitment

We aimed to recruit employee-manager pairs from businesses within the operational area of Burton and District Mind, located in the Midlands region (UK). Recruitment took place as part of the Mental Health & Productivity Pilot (MHPP) programme (https://mhpp.me/). Employer engagement and initial participant recruitment were jointly led by National Mind (JS) and a MHELW at Burton and District local Mind.

Following employers’ agreement to participate, wellbeing events were co-organised and delivered by the MHELWs. These events were promoted via internal employer communication channels using flyers and email circulars. Topics included stress management, positive psychology, and strategies for promoting mental health and engagement at work. Events were co-designed by the MHPP team, local Mind staff, and the lead academic psychologist (AP), with the dual aim of raising mental health awareness and encouraging participation in MENTOR 2.0.

Three MHELWs were trained by the MHPP team using the original MENTOR 1.0 training framework [see 13]. The training programme was refined for this feasibility study, reduced from 50 to 26 h delivered over two weeks. This modification was intended to enhance feasibility and scalability while preserving intervention fidelity. Training content included mental health literacy, employee engagement, communication skills, and intervention protocol adherence. Training sessions were co-delivered by Mind and the lead academic.

In addition to intervention delivery, MHELWs were trained in employer engagement and participant recruitment strategies. During each employer event, MHELWs provided information about MENTOR 2.0 and invited attendees to express interest in participating. Ultimately, three employers took part in the recruitment process, and one employer from the hospitality sector consented to full participation in the study.

Although the recruitment strategy was designed to attract a diverse sample across multiple industries, the final sample was drawn exclusively from the hospitality sector.

### Participants

A total of 24 employee–manager pairs were recruited to take part in the MENTOR 2.0 intervention. Of these, one employee decided to participate without their manager’s involvement. This resulted in a final sample of 23 employees and 19 managers, including four managers who supported two separate employees each. An overview of participant flow and reasons for withdrawal is presented in Fig. 2.

Table 1 outlines the inclusion and exclusion criteria. A key distinction between MENTOR 1.0 and MENTOR 2.0 was the removal of a formal mental health diagnosis as an eligibility requirement. This change was made in response to stakeholder feedback, grounded in both ethical and practical considerations. The diagnostic criterion was viewed as a potential barrier to participation and a mechanism that could inadvertently reinforce stigma. Its removal was intended to enhance accessibility and support early, preventative intervention in line with contemporary models of workplace mental health promotion (Table 1).

**Table 1:** MENTOR 2.0 participants’ inclusion and exclusion criteria

Inclusion criteria	Exclusion criteria
Participants must be 18 + years of age Must be able to give consent Must be willing to work with a MHELW and their business line manager	Employees currently on sick leave Currently in an acute mental health crisis as reported by themselves Currently on extended sick leave Receiving input from an individual placement and support (IPS) worker and planning to retire within the next 12 months and unable to complete intervention and evaluation

### MENTOR 2.0 intervention

MENTOR 2.0 is an adapted version of the original MENTOR 1.0 intervention [13], developed to promote psychological wellbeing and employment sustainability among workers experiencing mental health difficulties. The intervention was preregistered in the ISRCTN registry on 20/12/2023 (ISRCTN79256498).

MENTOR 1.0 was co-designed through three stakeholder workshops involving employees with lived experience of mental health challenges, line managers, and operational staff from Midlands-based branches of the mental health charity Mind. Additionally, mental health practitioners were consulted via interviews to refine the intervention content and delivery format. The resulting intervention was delivered via Zoom by MHELWs, who received 50 h of training. MENTOR 1.0 included ten sessions: four joint employee–manager meetings, three employee-only sessions, and three manager-only sessions. It focused on three core themes:

- Staying well and being productive at work (work engagement),
- Having open conversations about mental health (interpersonal relationships),
- Managing difficult situations at work (psychological flexibility).

**Fig. 1:**
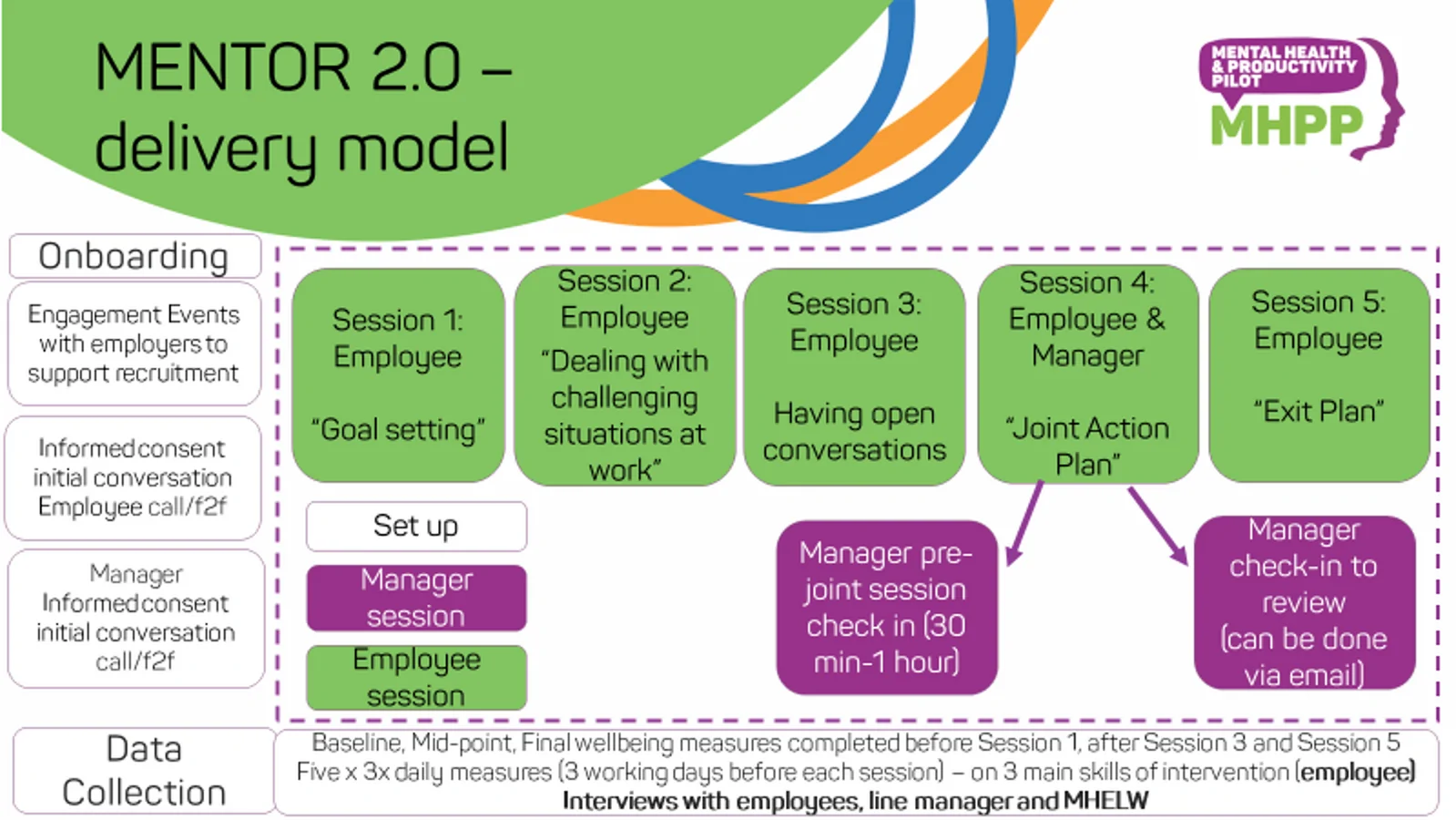
MENTOR 2.0 delivery model

Drawing on findings from the MENTOR 1.0 feasibility study [1], the Employment Liaison Worker Pilot (a separate independent pilot led by Mind), and additional feedback from focus groups and interviews, the MENTOR 2.0 model was refined to improve feasibility, reduce participant burden, and enhance scalability (see Fig. 1). Key adaptations included:

- A reduction in the number of sessions from ten to five, while retaining the core thematic content through an integrated delivery format.
- A streamlined role for managers, involving:

A 30-minute introductory session,

An optional 30-minute preparatory session with the MHELW,

One joint employee–manager session,

An optional 30-minute follow-up meeting.

MENTOR 2.0 comprised the following sessions:

- Session 0: Introductory one-to-one meeting between the MHELW and the employee (overview of the study and eligibility screening),
- Session 1: Goal setting and values clarification,
- Session 2: Managing challenging situations at work,
- Session 3: Facilitating open conversations about mental health,
- Session 4: Joint action planning session with the manager,
- Session 5: Exit planning and sustainability.

All sessions were delivered online by three trained MHELWs. Although the programme was designed to be delivered weekly, a flexible 12-week window was allowed to accommodate participant availability, leave, or sickness absence. The training programme for MHELWs was reduced from 50 to 26 h (over two weeks) to enhance feasibility while maintaining fidelity. Training content covered core areas, including mental health literacy, communication skills, employee engagement, and intervention protocol delivery. These changes reflect an iterative development approach grounded in stakeholder feedback and implementation science principles.

### MENTOR intervention logic model

To articulate the theoretical foundations and operational structure of the MENTOR intervention, a logic model was developed (see *Supplementary Fig. 1*). This model visually represents the hypothesised pathway through which the intervention’s resources, activities, and intermediate processes are expected to produce short-, medium-, and long-term outcomes for employees and managers.

MENTOR intervention was co-designed with input from employees with lived experience of mental health challenges, line managers, and mental healthcare practitioners. The model addresses key barriers faced by employees with mental health conditions in sustaining engagement and productivity at work, while also supporting managers in fostering psychologically safe and inclusive work environments.

### Procedure

All interested employees and managers received a detailed Participant Information Sheet. Informed consent was obtained via a secure digital form hosted on the Qualtrics survey platform. Upon providing consent, participants were asked to complete a demographic questionnaire, a baseline survey, and three consecutive daily surveys prior to the first intervention session (baseline daily surveys).

Throughout the intervention period, participants received three consecutive daily surveys following each of the five sessions (15 in total), in addition to a mid-intervention survey administered after the third session. After completing the final session, participants were asked to complete a post-intervention survey and a qualitative feedback form to evaluate their experience.

Participating managers completed a separate informed consent form, a demographic questionnaire, and baseline and post-intervention surveys, along with a qualitative feedback form at the end of the programme.

To support engagement and reflection, employees received structured participant workbooks aligned with the content of each session. These workbooks were designed to enhance learning, support goal setting, and allow space for personal reflection. Managers were also provided with a ‘useful tips’ booklet, which offered practical guidance on how to support employees during and after the intervention. MHELWs further supplemented sessions with wellbeing tools and supported self-help resources, such as stress management strategies and goal-setting exercises.

### Outcome measures

#### Baseline, mid-intervention, and post-intervention surveys (employees)

Employees completed surveys at three primary time points: baseline (prior to Session 1), mid-intervention (following Session 3), and post-intervention (following Session 5). The following measures were used to assess psychological health outcomes and hypothesised intervention mechanisms:

##### Psychological distress

Psychological distress was assessed using the 12-item General Health Questionnaire (GHQ-12) [14], a validated and widely used measure in occupational mental health research [15]. Items are rated on a 4-point Likert scale, with total scores ranging from 0 to 36. Higher scores indicate greater distress. GHQ-12 thresholds were interpreted as follows: <10: Asymptomatic, 11–14: Sub-symptomatic, 15–19: Symptomatic, 20–36: Highly symptomatic.

##### Productivity

A single-item measure of perceived daily productivity was used [16], asking participants to rate their productivity as a percentage from 0% (very low/none) to 100% (very high productivity). This pragmatic measure was selected to minimise burden and capture workplace-relevant functioning.

##### Work engagement, psychological flexibility and interpersonal relationships

These were included to assess MENTOR processes and mechanisms of change using the following measures:

##### Work engagement

Work engagement was assessed using three items adapted from the Short Work Engagement Scale [17], measuring enthusiasm, inspiration, and pride in one’s work (e.g., “I am enthusiastic about my job”).

##### Psychological flexibility

To assess psychological flexibility, the Valued Action subscale of the CompACT measure was used [18]. This subscale includes 8 items evaluating individuals’ ability to engage in goal-directed action aligned with personal values (e.g., “I am able to follow my long-term plans, including times when progress is slow”).

##### Interpersonal relationships at work

Perceptions of workplace relationships were assessed using the Work Relations Scale developed by Biggs and colleagues [19]. This scale captures key relational dynamics between employees and their line managers or supervisors (e.g., “I am valued by my manager”).

##### Quality of employee-manager relationship

As a post-intervention measure, employees rated the perceived quality of their relationship with their manager using a single, ad-hoc item on a 7-point Likert scale (1 = very poor, 7 = excellent). This item was intended to provide a brief, subjective indicator of interpersonal outcomes resulting from the joint session.

#### Manager measures

Managers completed a baseline survey before their employee began the intervention and a post-intervention survey after the employee’s final session. These included:

##### Interpersonal relationships

Measured using the seven-item Leader–Member Exchange scale (LMX-7) [20], adapted by replacing the term follower with employee. This scale assesses the quality of the employee–manager relationship across dimensions such as mutual respect, trust, and reciprocal obligation.

##### Demographics

Managers provided demographic information, including age, gender, ethnicity, occupational role, and any self-reported mental health conditions.

##### Daily measures (Employees)

As part of the feasibility design, employees were asked to complete daily evening surveys throughout the intervention period. These brief surveys captured real-time fluctuations in psychological outcomes and potential mechanisms of change, and included the following domains:

##### Intervention adherence

Adherence to the intervention was monitored by the MHELWs throughout the study period. MHELWs recorded the number of sessions attended, session duration, cancellations, and rescheduled sessions using an intervention adherence tracker provided by the mental health charity Mind. In cases of withdrawal or dropout, MHELWs followed up with participants to document the reason, where possible. This tracking process facilitated systematic case management and monitoring of participant progress across the intervention period.

#### Psychological outcomes (Employees)

##### Mood

To assess mood, six items from the Positive and Negative Affect (PANAS) scale [21] were used. Three items assessed depressive symptoms, and three items assessed enthusiasm. Participants were asked to indicate how they felt that day on a scale from 1 (very slightly or not at all) to 5 (extremely).

##### Perceived stress

Participants were asked to complete four out of ten items from the Perceived Stress scale [22]. These items were chosen as they focus on the individual’s perceived control over their day. An example of the item was “Today, how often have you felt difficulties were piling up so high that you could not overcome them?”, rated from 0 (never) to 4 (very often).

##### Productivity

A single ad-hoc item was used to assess perceived productivity [16]. Participants rated their productivity levels (“How productive were you today?”) for the day in terms of percentage (0% = very low/none, 100% = very high productivity).

#### Active ingredients/psychological processes (Employees)

These measures were chosen to assess hypothesised mechanisms of change within the MENTOR model:

##### Work engagement

Daily work engagement was assessed with an ad-hoc item: “Today I felt engaged, energised, enthusiastic in my daily activities at work” (1 = Not at all, 7 = Very Engaged).

##### Interpersonal relationships at work

Interpersonal relationships were assessed with two items adapted for daily use from the Work Relations Scale [19]: “Today some co-workers were hard to work with” and “Today I found it hard to work with my manager” (1 = Strongly Disagree, 7 = Strongly Agree).

##### Psychological flexibility

Three items were selected from the CompACT scale and used for the daily surveys [18]. Items were selected due to being part of the ‘valued action’ subscale, which was chosen as it focuses on ‘action’ in line with MENTOR’s mechanisms of the intervention. The items were: “Today I behaved in line with my personal values”, “Today I could identify the things that really matter to me in life and pursue them” and “Today my values were really reflected in my behaviour” (0 = Strongly Disagree, 6 = Strongly Agree).

##### Mental health awareness

Mental Health Awareness was assessed with an item created ad-hoc: “Today/Over the past 2 weeks, how open, willing, and confident did you feel to talk about your daily activities and/or mental health with your manager?” (1 = Not open, willing, confident, 7 = Extremely high open, willing, confident).

#### Post-intervention feedback

Both employees and managers were asked to provide qualitative feedback after completing their post-intervention survey to capture acceptability, perceived usefulness, and suggestions for improvement. Employees and managers completed separate questionnaires via Qualtrics.

##### Qualitative feedback (Employees)

This consisted of four open-ended items aimed at understanding participants’ overall experience and learnings from MENTOR 2.0.

##### Qualitative feedback (Managers)

This consisted of three open-ended items aimed at examining managers’ perspectives on MENTOR 2.0 and any observed changes in their employees.

### Data analyses

All quantitative analyses were conducted using IBM SPSS Statistics Version 29 (IBM Corp., 2022). Given the feasibility nature of the study, the primary focus was on evaluating feasibility and acceptability outcomes. However, preliminary exploratory analyses were also performed to examine within-subject changes over time on key psychological and work-related variables. Due to the small sample size and non-randomised design, all statistical analyses beyond descriptive reporting - including repeated measures and moderation models - were considered exploratory and hypothesis-generating, rather than confirmatory.

Descriptive statistics (means and standard deviations) were calculated to summarise outcomes at baseline, mid-intervention, and post-intervention for both employees and managers. An intention-to-treat (ITT) approach was employed, and multiple imputation procedures were used to address missing data points, assuming data were missing at random.

To assess changes over time, repeated measures ANOVAs were conducted with Time (three levels: baseline, mid-intervention, post-intervention) as the within-subjects factor. The assumption of sphericity was tested for all outcome variables using Mauchly’s test. For variables where this assumption was violated - including psychological distress, work engagement, and employee–manager relationship quality - Greenhouse–Geisser corrections were applied.

### Multilevel modelling analysis

Multilevel modelling (MLM) analyses were conducted using Hierarchical Linear Modelling software (HLM, Version 8) [23], which allows for the examination of nested data structures, such as repeated measures (Level 1) nested within individuals (Level 2). These analyses aimed to address Aim 2 (changes in state psychological outcomes and processes) and Aim 3 (moderation by baseline psychological distress and mental health diagnosis). At Level 1, within-person state variables were modelled across time using data from the daily surveys, including perceived stress, mood, productivity, work engagement, mental health awareness, interpersonal relationships at work, psychological flexibility. At Level 2, between-person trait-level predictors included: baseline psychological distress, trait productivity, work engagement, psychological flexibility, interpersonal relationships, quality of the employee–manager relationship.

In line with best practices in MLM [e.g., 24], Level 1 variables were group-mean centred, and Level 2 variables were grand-mean centred. The primary objective of these analyses was to assess whether participation in MENTOR 2.0 was associated with systematic changes over time in daily psychological outcomes and process variables (Level 1 slopes), indicating short-term improvements in key constructs during the intervention period. Additionally, cross-level interactions were tested to evaluate whether baseline psychological distress and the presence of a mental health condition (Level 2 variables) moderated change trajectories in state-level outcomes, in line with Aim 3.

Given the feasibility nature of the study, these MLM results are considered exploratory and intended to inform the design and hypotheses of future, fully powered trials.

## Results

### Demographic information

#### Employees

Of the 23 employees enrolled in the study, 17 participants (74%) completed the demographic questionnaire. Among these respondents, 10 identified as female (59%) and 7 as male (41%). The mean age was 36 years (range: 23–58). All participants were recruited from the same organisation within the hospitality sector.

In terms of ethnicity, 16 participants (94.1%) identified as White British, and 1 participant (5.9%) identified as Other White background. Regarding mental health status, 4 participants (23.5%) reported having a formal diagnosis of a mental health condition, all of which were anxiety-related disorders. One participant (5.9%) preferred not to disclose this information.

#### Managers

Of the 19 managers who took part in the intervention, 12 (63%) completed the demographic questionnaire. Of these, 9 identified as female (75%) and 3 as male (25%). The mean age was 41 years (range: 30–52). All manager respondents identified as White British. No managers reported having a diagnosed mental health condition.

**Fig. 2:**
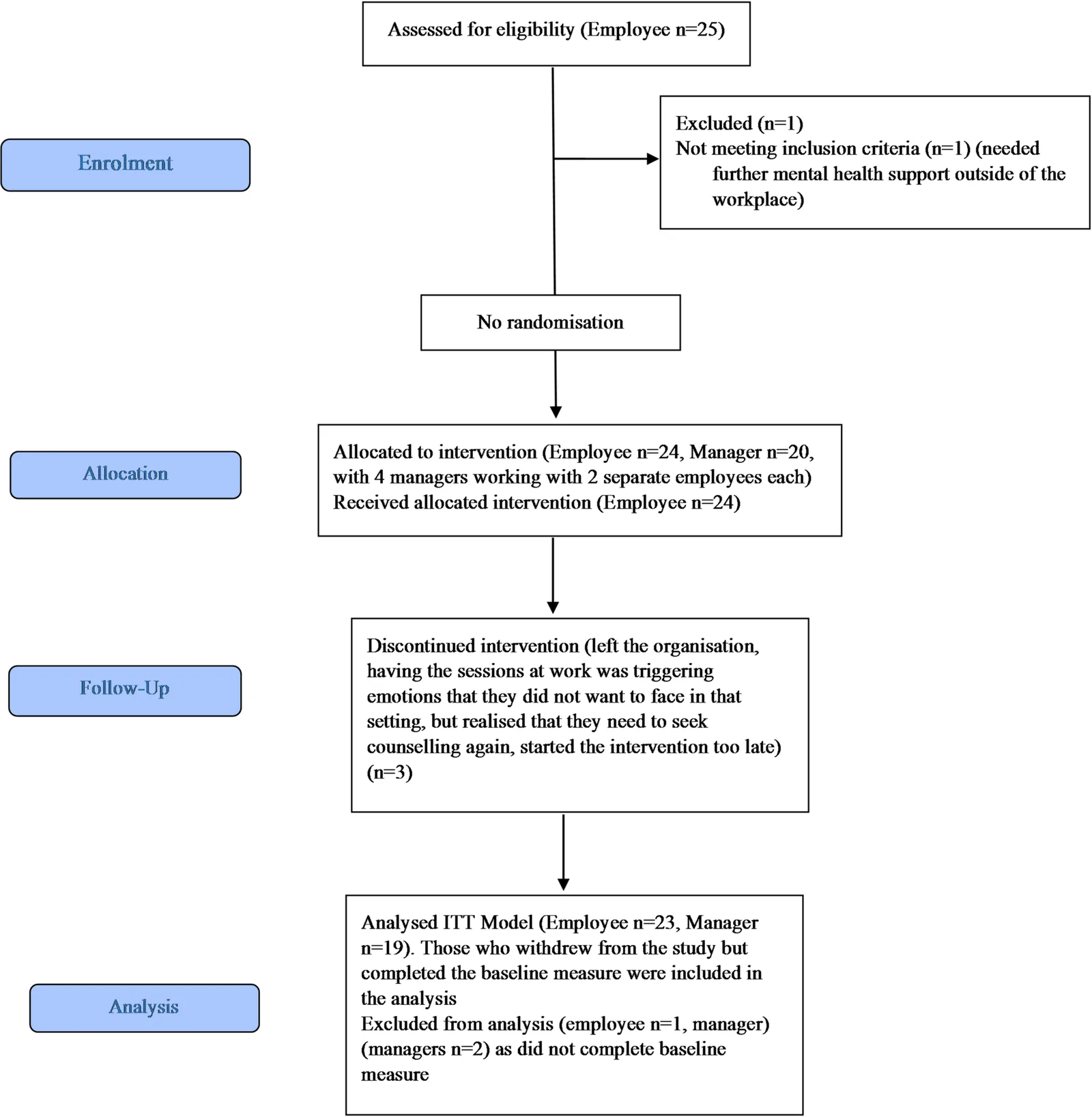
CONSORT 2010 Flow Diagram

### Aim 1: to assess the acceptability and feasibility of MENTOR 2.0

A total of 108 intervention sessions were delivered across participants, with 94% (*n* = 102) completed on the originally scheduled date and time. Six sessions (5.6%) were cancelled due to participants’ limited availability, commonly related to workplace demands or illness. The average duration to complete the five-session intervention (Sessions 1–5) was 65 days, reflecting moderate flexibility in scheduling. 91% of sessions lasted at least the intended 50 min, indicating strong adherence to the planned session structure. A total of 16 managers participated, four of whom supported two employees each, highlighting the intervention’s flexibility to accommodate supervisory roles with multiple direct reports. Three employees withdrew from the intervention after completing the baseline assessments. Reasons for discontinuation are detailed in Fig. 2, with common factors including competing responsibilities and reduced capacity to engage.

These adherence patterns suggest that MENTOR 2.0 was feasible to deliver in a real-world workplace context. However, instances of withdrawal and session rescheduling highlight the need for ongoing support and flexible delivery options to maintain engagement, particularly in high-demand sectors such as hospitality.

### Preliminary analyses

Participants who completed a baseline assessment were included in both Level 1 (daily measures) and Level 2 (trait-level measures) datasets. One employee and two managers who did not complete baseline assessments were excluded from the analyses. Outliers were identified using boxplots, histograms, and Mahalanobis distance. Where significant outliers were detected, winsorization was applied - extreme values were replaced with the nearest non-outlier value to reduce undue influence on the results.

All variables demonstrated approximate normality, with skewness and kurtosis values within the acceptable range (± 1.0), and confirmed through visual inspection of histograms. To assess the pattern of missing data, Little’s Missing Completely at Random (MCAR) test was conducted:

The employee Level 2 dataset (χ² = 15.31, df = 18, *p* =.64) and manager Level 2 dataset (χ² = 1.26, df = 1, *p* =.26) showed no significant departures from randomness, suggesting data were missing completely at random (MCAR). However, the employee Level 1 dataset (χ² = 30.68, df = 9, *p* <.001) indicated that missing daily data were not MCAR, suggesting a systematic pattern in non-response.

To address missing data, multiple imputation was performed following established best practices [24, 25]. Ten imputed datasets were generated and pooled for analysis. Imputation was conducted at the composite score level, a method considered appropriate for small sample sizes with relatively high missingness [26, 27]. For the repeated measures ANOVA analyses, the assumption of sphericity was met for most outcome variables. Where violations were observed - specifically for psychological distress, work engagement, and quality of the employee–manager relationship - Greenhouse–Geisser corrections were applied.

### Descriptive statistics

#### Employees

Descriptive statistics for employee outcomes at baseline, mid-intervention, and post-intervention are presented in Table 2. At baseline, participants scored an average of 15.78 (SD = 4.72) on the GHQ-12, indicating ‘symptomatic’ levels of psychological distress. By post-intervention, mean GHQ-12 scores had decreased to 6.35 (SD = 3.55), a level categorised as ‘asymptomatic’, suggesting a substantial reduction in self-reported distress over the course of the intervention.

Positive trends were also observed across all other primary outcomes. Positive improvements were found in psychological flexibility (as measured by the Valued Action subscale), interpersonal relationships at work (as measured by the Work Relations Scale), work engagement, and self-reported productivity. Although several of these changes reached statistical significance in repeated measures analyses, the results should be interpreted with caution given the single-arm feasibility design, small sample size, absence of a control group, and the use of self-report measures.

Nevertheless, the observed effect sizes and consistent direction of change across measures offer encouraging preliminary evidence for the potential psychological and workplace benefits of the MENTOR 2.0 intervention.

Full descriptive and inferential results are provided in Table 2.

**Table 2:** Intention-to-treat (ITT) baseline, mid and Post-intervention means and standard deviations (*n* = 23)

	Baseline (Pre-Session 1) M (SD)	Mid-Intervention (Post-Session 3) M (SD)	Post-Intervention (Post-Session 5) M (SD)	F-Statistic
Psychological Distress	15.78 (5.95)	9.09 (4.81)	6.35 (3.55)	*F*(1.30, 28.61) = 27.80, *p* =.001
Productivity	3.6 (0.79)	3.43 (1.03)	4.05 (0.58)	*F*(2, 44) = 5.28, *p* =.002
Work Engagement	13.44 (2.61)	14.17 (2.30)	14.8 (2.20)	*F*(1.34, 29.49) = 9.78, *p* =.001
Psychological Flexibility	33.39 (8.75)	38.00 (4.46)	40.06 (6.55)	*F*(2, 44) = 8.92, *p* =.001
Interpersonal Relationships at Work	38.96 (6.27)	40.91 (4.97)	42.27 (4.95)	*F*(2, 44) = 3.46, *p* =.02
Quality of Employee-Manager Relationship	5.8 (0.90)	5.91 (0.84)	6.06 (0.70)	*F*(1.49, 32.68) = 1.80, *p* =.178

#### Managers

Descriptive statistics for manager-rated outcomes at baseline and post-intervention are presented in Table 3. A repeated measures ANOVA was conducted to assess change in the perceived quality of employee–manager relationships, as measured by the Leader–Member Exchange (LMX-7) scale.

Results showed a statistically significant increase in LMX-7 scores from baseline (M = 26.00) to post-intervention (M = 27.97), F(1, 94) = 26.89, *p* =.001. Post-hoc pairwise comparisons, using a Bonferroni correction, confirmed that this increase was significant (*p* =.001), suggesting a meaningful improvement in managers’ perceptions of their relationships with employees following participation in MENTOR 2.0.

**Table 3:** Manager baseline and post-intervention means and standard deviations (*n* = 19)

	Baseline M (SD)	Post M (SD)	F-Statistic
LMX-7	26 (3.30)	27.97 (4.21)	F(1,94) = 26.89, *p* =.001

### Aim 2: changes in psychological and work-related outcomes (Employees)

**Fig. 3:**
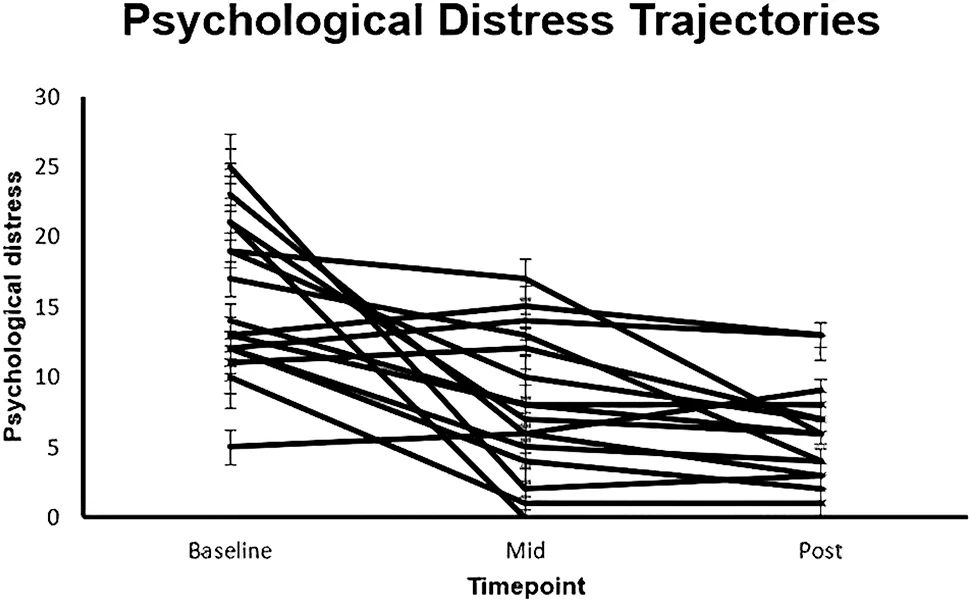
Individual-level changes in psychological distress by participants from baseline to mid-intervention and post-intervention assessments.

A series of repeated measures ANOVAs were conducted to assess changes in psychological and work-related outcomes across the three time points (baseline, mid-intervention, post-intervention). Results are summarised in Table 2 and visualised in Fig. 3.

Psychological distress (GHQ-12) decreased significantly over time, from M = 15.78 at baseline to M = 9.09 at mid-intervention and M = 6.35 at post-intervention, *F*(2, 44) = 27.80, *p* =.001. This represents a shift from ‘symptomatic’ to ‘asymptomatic’ levels, suggesting substantial improvements in emotional wellbeing over the course of the intervention. Productivity increased significantly between mid-intervention (M = 3.43) and post-intervention (M = 4.05), *p* =.002. However, this measure was self-reported and may be subject to bias or overestimation. Work engagement improved significantly from M = 13.43 at baseline to M = 14.80 at post-intervention, *p* =.001, indicating enhanced enthusiasm and involvement in work tasks. Psychological flexibility, measured via the Valued Action subscale, increased from M = 33.40 at baseline to M = 40.10 at post-intervention, *p* =.001, suggesting greater alignment between daily behaviour and personal values. Interpersonal relationships at work also showed a statistically significant improvement, increasing from M = 39.00 to M = 42.27, *p* =.02, reflecting strengthened workplace social dynamics. In contrast, there was no statistically significant change in the quality of the employee–manager relationship, *F*(1.49, 32.68) = 1.80, *p* =.178.

#### Hierarchical linear modelling results

Given the small sample size and the exploratory nature of this feasibility study, all multilevel findings - particularly moderation effects - should be interpreted with caution. Full model outputs are available in the Supplementary Materials.

##### Aim 3: changes in state psychological health and processes across the intervention

To address Aim 3, HLM analyses were conducted to examine whether participation in MENTOR 2.0 was associated with change over time in state psychological health (perceived stress, mood, job satisfaction, wellbeing, work engagement, and productivity) and hypothesised state psychological processes (psychological flexibility, mental health awareness, interpersonal relationships at work, and work engagement). These Level 1 models captured within-person variation across the intervention period. Results are summarised in Table 4.

Despite the limited statistical power, several significant relationships emerged between state psychological outcomes and daily process variables. There was a negative association between stress and the state psychological processes of daily work engagement (β = −0.316, *p* =.018), interpersonal relationships at work (β = −0.280, *p* <.001), and psychological flexibility (β = −0.212, *p* <.001). In other words, lower stress levels were linked to higher work engagement, better interpersonal relationships, and greater psychological flexibility. The HLM analyses also found that higher positive affect was significantly associated with greater levels of psychological flexibility (β = 0.362, *p* <.001). Conversely, greater negative affect was significantly associated with lower scores in interpersonal relationships (β = −0.404, *p* <.001) and psychological flexibility (β = −0.132, *p* =.003). Finally, higher self-reported productivity was significantly associated with greater levels of psychological flexibility (β = 0.058, *p* <.001). See Table 4 for a summary of results.

##### Aim 4: moderation by baseline psychological distress and diagnosed mental health condition

To address Aim 4, cross-level moderation analyses were conducted to examine whether baseline psychological distress or the presence of a formally diagnosed mental health condition (Level 2 variables) moderated associations between daily state variables (Level 1).

Preliminary indications from these analyses suggested that higher levels of baseline psychological distress appeared to modify the strength of the association between daily perceived stress and mental health awareness (β = 0.041, *p* =.044). Similarly, baseline psychological distress seemed to influence the relationship between daily positive affect and both interpersonal relationships (β = −0.028, *p* =.002) and psychological flexibility (β = 0.020, *p* =.044). The presence of a formally diagnosed mental health condition at baseline did not emerge as a significant moderator of these state-level relationships in this exploratory analysis (all *p* >.05).

Due to the limited sample size and exploratory nature of the study, these findings are not sufficiently powered for confirmatory conclusions. See Supplementary Tables 1 and 2 for moderation analyses.

**Table 4:** Level 1 models

Perceived Stress	β	Coefficient	SE	*p*-value	Positive Affect	β	Coefficient	SE	*p*-value	Negative Affect	β	Coefficient	SE	*p*-value	Productivity	β	Coefficient	SE	*p*-value
Intercept	*β* *00*	9.897	0.102	< 0.001		*β* *00*	9.365	0.118	< 0.001		*β* *00*	5.714	0.095	< 0.001		*β* *00*	3.907	0.030	< 0.001
Work Engagement	*β* *10*	−0.316*	**0.133**	**0.018**		*β* *10*	0.186	0.154	0.229		*β* *10*	0.023	0.124	0.856		*β* *10*	0.265	0.038	< 0.001
Mental Health Awareness	*β* *20*	−0.043	0.123	0.723		*β* *20*	0.092	0.142	0.517		*β* *20*	−0.101	0.114	0.376		*β* *20*	−0.057	0.035	0.106
Interpersonal Relationships	*β* *30*	−0.280**	**0.060**	**< 0.001**		*β* *30*	0.106	0.070	0.130		*β* *30*	−0.404**	**0.056**	**< 0.001**		*β* *30*	−0.001	0.017	0.955
Psychological Flexibility	*β* *40*	−0.212**	**0.048**	**< 0.001**		*β* *40*	0.362**	**0.056**	**< 0.001**		*β* *40*	−0.132*	**0.045**	**0.003**		*β* *40*	0.058**	**0.014**	**< 0.001**

** *p* <.001, * *p* <.05

Bolded coefficients and β values denote statistically significant predictors at *p* < .05

* indicate levels of significance (*p < .05; *p < .01)

#### Qualitative results

Post-intervention qualitative feedback was collected from both employees and managers via open-ended questionnaires. A reflexive thematic analysis approach was used to identify patterns in participants’ responses [28]. All identifying information was anonymised prior to analysis. Thematic development was inductive and aimed at understanding participants’ experiences of the MENTOR 2.0 intervention, particularly its perceived acceptability and impact. Key themes from the employee data are presented below, supported by illustrative quotes.

#### Employee feedback

##### What was the best part of MENTOR?

**Open and honest conversations**

When asked about the most helpful part of the intervention, participants described the value of being able to have “open and honest” conversations. The one-to-one sessions were highlighted as providing a comfortable space to express personal concerns, whether related to “work-life” or “anything at all.”

**Rapport with the MHELW**

Another common theme was the relationship developed with the MHELW. Participants identified this as one of the best aspects of MENTOR, specifically noting the MHELW’s “approach, understanding, and manner.” One participant shared that they felt “really listened to,” and appreciated that “someone understood my concerns and was able to give me advice relevant to my mindset.”

**Self-Reflection**

A further theme related to self-reflection and insight. Participants reported that MENTOR helped them to better understand their own thoughts, behaviours, and response patterns. Having a “designated time to reflect” allowed participants to “take a step back” from busy work routines and engage in “looking internally” and “self-evaluation.”

**Describe your experience of working with an MHELW**

Participants reported consistently positive experiences of working with a MHELW. No negative feedback was recorded in response to this question.

A strong theme of trust and confidentiality emerged, with participants describing how sessions with a MHELW allowed them to speak freely about workplace issues. Several participants emphasised the value of having a neutral third party, unaffiliated with their employer. One participant noted: “I didn’t have to worry about impacting working relationships as they (the MHELW) were completely unrelated to the company.”

The presence of the MHELW was reported to facilitate open and honest dialogue, with one participant stating:

“[They] made me feel very comfortable talking about things that I was really unsure of talking about.”

Participants also highlighted the practical and emotional support provided during sessions. MHELWs were described as offering both relevant tools to support wellbeing and compassionate understanding. As one employee explained:

“My liaison worker has been excellent through this process. She has been really supportive and understanding of the situation and has given me lots of helpful tools to support myself and also my team. She has been excellent and I am really grateful for everything she has done for me, making me feel very comfortable.”

These responses underline the perceived value of the MHELW role as a key element in the intervention’s acceptability and impact.

**What do you think could be improved about MENTOR 2.0?**

Three key themes emerged from participants’ responses regarding potential improvements to the MENTOR 2.0 programme. First, participants commented on the burden of completing multiple surveys, particularly the daily measures administered after each session. These were described as tedious and repetitive, which may have affected engagement with the study protocol. Although these surveys were not part of the MENTOR 2.0 intervention itself but rather part of the research data collection, the feedback offers valuable insight into the acceptability of the study methodology.

Second, some participants raised concerns about the usability of the workbooks provided alongside sessions. While the workbooks were designed to enhance reflection and reinforce session content, several participants found them difficult to follow independently. They expressed a preference for working through the materials during the sessions, rather than completing them alone in their own time.

Third, a strong theme emerged around the desire for ongoing support following the conclusion of the programme. Several participants suggested the inclusion of follow-up sessions to monitor progress and reinforce the benefits of the intervention. One participant described this as a potential “Stage 2: Follow-Up”, while another suggested future check-ins “to see if things are still on track”. These suggestions point to clear avenues for refinement - both in terms of research design (e.g. reducing participant burden from surveys) and intervention structure (e.g. workbook integration and post-programme support) - and may enhance the long-term impact and sustainability of MENTOR 2.0.

**Is there anything you can do now, that you could not do before the MENTOR 2.0 intervention?**

Participants reported a range of new skills and personal developments that they attributed to their involvement in the MENTOR 2.0 intervention. Three key themes emerged from responses to the question: “Is there anything you can do now that you could not do before the MENTOR 2.0 intervention?”

First, participants described an increased ability to communicate more openly and constructively in the workplace. Several employees noted they had become more comfortable discussing personal or work-related difficulties. For example, participants said they had become “more open and honest about how [they] were feeling” and had “better open conversations with both [their] boss and others within [their] team.” The intervention appeared to support individuals in “approaching people with personal issues” and “sharing problems to make them seem like less of a problem.”

Second, a theme of enhanced self-confidence was evident. Participants reported feeling more secure in their work-related abilities and less self-critical. One individual described learning to “stop being so hard” on themselves, while another said they now believed they were “good enough.” These changes were linked to reductions in anxiety and a greater sense of ease in the workplace.

Third, participants identified changes related to productivity and goal-setting. One person reported feeling “more productive” since completing the programme, attributing this to the use of specific strategies introduced during MENTOR. Another reflected on a shift away from a narrow focus on task-driven output, towards a more balanced view of productivity that included wellbeing and personal effectiveness.

Together, these themes suggest that the MENTOR 2.0 intervention may foster meaningful behavioural, emotional, and interpersonal change, enhancing participants’ ability to navigate workplace challenges with increased self-awareness and confidence.

#### Managers’ perspectives on MENTOR 2.0

##### How much do you feel this is related to taking part in the intervention together?

Managers were also asked how much they felt the changes observed were related to participating in the intervention together. While some comments focused on the broader benefits of the intervention, one manager explicitly stated: “The joint session definitely helped.” Another manager reflected more generally, noting: “The scheme was the trigger to drive change.” These responses suggest that, even with limited direct involvement, the joint component of MENTOR 2.0 may have symbolic value and help reinforce the relational and behavioural shifts fostered by the intervention.

#### Would you have wanted more/less sessions?

Managers were divided in their views on the number of sessions. 58% felt that the number of sessions was appropriate, with some stating that even one session was “powerful enough.” 16% expressed a preference for fewer sessions, though no additional rationale was provided. 26% indicated that additional sessions, particularly follow-up meetings, would be helpful to sustain change over time. A recurrent suggestion was the need for greater clarity and structure regarding the manager’s role in the joint session. One manager commented: “I felt slightly blind going into the session; I wasn’t aware of goals and what they [the employee] were working on.” This feedback points to a need for better preparation and communication ahead of the joint meeting, to ensure that both parties are aligned and equipped to engage meaningfully.

#### Overall, have you noticed a change in your employee?

Of the managers who completed the qualitative questionnaire, 91% reported noticing a positive change in their employee following participation in MENTOR 2.0. Managers most frequently noted improvements in employee confidence, with one describing “a big change” in how their employee presented at work. Interestingly, the one manager who did not perceive a change in their employee stated: “I just understand them better now, so the change is more on my part.” This suggests that even in the absence of observable behavioural change, the intervention may support improved manager insight and empathy.

## Discussion

This study assessed the feasibility and acceptability of the adapted MENTOR 2.0 intervention for employees, while also exploring its potential to improve psychological health among employees, both with and without mental health conditions. The findings suggest improvements in psychological health outcomes over the course of the intervention; however, given the feasibility design and absence of a control group, these results should be considered preliminary.

The high adherence rates, minimal session cancellations, and strong retention beyond baseline indicate that MENTOR 2.0 was feasible to deliver in a real-world workplace context. The flexibility in scheduling (mean completion time: 65 days) and low dropout rate demonstrate the practicality of embedding such an intervention within employee wellbeing programmes. These outcomes are consistent with previous research emphasising the value of structured yet adaptable workplace mental health programmes [6, 7]. One key challenge noted was time constraints due to work commitments, which contributed to attrition among three employees. Future iterations of MENTOR could explore shorter sessions or the inclusion of asynchronous elements (e.g., self-paced digital modules) to enhance accessibility amidst fluctuating workplace demands.

Quantitative results showed a significant reduction in psychological distress (as measured by GHQ-12), moving participants from symptomatic to asymptomatic ranges by the end of the intervention. This is a promising finding, particularly given the high initial levels of distress reported at baseline. Statistically significant improvements were also found in self-reported productivity, work engagement, psychological flexibility, and interpersonal relationships at work. These changes suggest that MENTOR 2.0 has potential to enhance both psychological wellbeing and workplace functioning. However, it is important to note that productivity was measured solely through self-report, which is subject to biases such as recall error and social desirability. Future research should aim to incorporate objective or multi-informant measures (e.g., supervisor ratings or performance data) to validate these preliminary findings.

Qualitative findings suggested that employees had highly positive experiences, particularly with the support provided by MHELWs. Participants emphasised the trust, rapport, and psychological safety fostered through these one-to-one relationships, which in turn facilitated meaningful engagement with the intervention content. The structured focus on self-reflection and practical problem-solving was also seen as a key benefit. These findings suggest that manager-employee mental health interventions may be more effective when combining personalised support with targeted, action-oriented strategies. However, it is important to interpret the positive qualitative feedback with caution. Factors such as sampling bias, self-selection, social desirability, and inherent power dynamics within workplace settings may have influenced participants’ responses. To mitigate these potential biases, future studies should consider incorporating independent evaluation procedures, as well as anonymous feedback mechanisms, to elicit more balanced perspectives.

Participants also provided constructive suggestions to enhance intervention acceptability. These included reducing the burden of frequent surveys, improving the clarity and structure of session workbooks, and introducing follow-up sessions to help maintain behavioural change. Addressing these recommendations in future trials may further improve participant experience and intervention outcomes.

Interestingly, there was a discrepancy between managers’ and employees’ perceptions of improvements in their relationships. While most managers reported seeing positive changes, this was not mirrored by significant improvements in the employee-reported quality of the employee-manager relationship. It is possible that managers’ perceptions reflected increased awareness or empathy, whereas employees may have expected more visible behavioural changes. Additionally, the limited frequency of joint sessions and underlying hierarchical dynamics may have contributed to these differing perspectives. Future research should explore these relational dynamics in more depth, potentially increasing the number of joint sessions or clarifying mutual goals to strengthen alignment and impact.

With regard to psychological processes, multilevel modelling preliminary results revealed that decreases in stress were associated with increases in work engagement, interpersonal relationships, and psychological flexibility. Positive affect was positively associated with psychological flexibility, while negative affect was negatively associated with both interpersonal relationships and psychological flexibility. Baseline psychological distress emerged as a significant moderator for several of these relationships - for example, influencing the strength of associations between stress and mental health awareness, and between positive affect and interpersonal functioning. Notably, having a clinical mental health diagnosis did not moderate these effects, suggesting that the intervention may be beneficial for employees irrespective of formal diagnostic status.

The results of this study resonate with the growing body of research emphasising the importance of workplace interventions in improving mental health and overall wellbeing [7]. The preliminary reduction in psychological distress and improvements in psychological flexibility and interpersonal relationships observed in our study align with findings from Kinman and Clements [12] and LaMontagne et al. [6], who highlighted the important role of supportive workplace environments in mitigating mental health issues among employees. Our findings also extend the understanding of the role of manager-employee relationships in workplace mental health, underscoring the importance of managerial support as emphasised by Weinberg et al. [5] and LaMontagne et al. [6].

Furthermore, these initial outcomes parallel the objectives of the IAPT programme and IPS, reinforcing the potential of employer-based interventions like MENTOR 2.0 in addressing common mental health disorders. However, the mismatch in how managers and employees perceived relationship improvements underscores the complexity of workplace dynamics. Tailored approaches that attend to both perspectives are necessary, as suggested by Dimoff & Kelloway [9] and Duffy [10]. This study provides initial empirical support for multifaceted interventions like MENTOR 2.0, delivered within a workplace setting, potentially reducing reliance on already stretched healthcare services.

The study had some limitations. The small sample size and lack of a control group, along with the predominance of female participants from a single organisation, limit the generalisability of the findings. Both employees and managers expressed a desire for follow-up sessions, indicating a potential area for enhancement in future iterations of the programme. Additionally, the volume of required surveys (22 per participant) was perceived by some as overwhelming and repetitive, which may have impacted participant engagement and acceptability. Lastly, the study focused exclusively on employees within the hospitality sector - a field well known for its high levels of work-related stress. While this provides valuable insights, future research should consider diversifying recruitment strategies to include other high-stress but less accessible groups, such as legal professionals, accountants, those in professional services, as well as workers in manufacturing and construction.

The findings of this study offer several avenues for future research in workplace mental health interventions. Firstly, expanding the sample size and including a more diverse participant demographic across various industries would provide a broader understanding of the intervention’s applicability, helping to establish more robust evidence of its effectiveness. Secondly, future studies should explore the long-term effects of MENTOR 2.0 by including follow-up assessments several months post-intervention to assess sustainability. Thirdly, investigating which specific components of the intervention contribute most to observed improvements could enable refinement and greater scalability. Additionally, exploring the impact of cultural factors could inform adaptations of MENTOR 2.0 for diverse settings. Lastly, in light of the discrepancy observed between employees’ and managers’ perceptions of relationship change, future research should seek to better understand these dynamics and develop strategies to align managerial and employee expectations more effectively.

The preliminary improvements observed in psychological distress and other psychological outcomes underscore the potential value of interventions in the workplace. This suggests that organisations, particularly Small and Medium Enterprises (SMEs), could benefit from adopting structured mental health interventions into their employee wellness programmes. The role of MHELWs in delivering these interventions is particularly noteworthy, as they may provide a scalable and supportive avenue for in-house mental health care. Secondly, the disparity in perceptions of the employee-manager relationship improvement suggests a continuing need for manager training programmes that help managers understand and address the mental health needs of their employees. This is in line with the growing recognition of the manager’s role in fostering a supportive work environment and could lead to more effective communication and relationship-building strategies within organisations. Thirdly, the findings support multifaceted approaches to workplace mental health, aligning with the principles of the IPS programme, which emphasise not only clinical treatment but also education, workplace support, and resilience-building.

## Conclusions

Overall, the MENTOR 2.0 intervention shows promise as an approach for supporting psychological health in the workplace. The preliminary reductions in psychological distress and observed improvements in work-related outcomes provide a strong basis for further research. Future iterations of the programme could benefit from addressing the identified limitations, including broadening the participant pool, simplifying the workbook, reducing the survey burden, and incorporating follow-up sessions to sustain the intervention’s long-term benefits. Implementing these changes will help ensure that MENTOR 2.0 is both feasible and acceptable, ultimately contributing to more scalable and inclusive workplace mental health strategies.

## Supplementary Information

Below is the link to the electronic supplementary material.

Supplementary file1 (Docx 4265 KB)

## Data Availability

Access to raw participant data is restricted to protect confidentiality, but aggregate data and supplementary materials are available upon reasonable request. For access, please contact the corresponding author at a.prudenzi@sheffield.ac.uk.

## Appendix

### Appendix 1. Qualitative Feedback Questions

Employee Qualitative Feedback Questions:

1. What was the best part of MENTOR?
2. Describe your experience of working with an MHELW.
3. What do you think could be improved about MENTOR?
4. Is there anything you can do now, that you could not do before the MENTOR intervention?

Manager Qualitative Feedback Questions:

1. Overall, have you noticed a change in your employee?
2. How much do you feel this is related to taking part in the intervention together?
3. Would you have wanted more/less sessions?
